# Efficacy and safety of ultrasound-guided thermal ablation of graves’ disease: a retrospective cohort study

**DOI:** 10.1186/s13044-024-00198-4

**Published:** 2024-06-03

**Authors:** Guangzhen Cai, Beilin Luo, Maolin Wang, Jiqin Su, Luping Lin, Guibin Li, Xiangru Chen, Zhishu Huang, Peiyi Lin, Shengwei Liu, Huidi Yan, Lixin Zhou

**Affiliations:** 1 Department of General Surgery, The Second Affiliated Hospital of Xiamen Medical College, 566#, Shengguang Road, 361021 Xiamen, P.R. China; 2 The Graduate School of Fujian Medical University, The Second Affiliated Hospital of Xiamen Medical College, 88#, Jiaotong Road, 350005 Fuzhou, P.R. China; 3 Department of Endocrinology, The Second Affiliated Hospital of Xiamen Medical College, 566#, Shengguang Road, 361021 Xiamen, P.R. China

**Keywords:** Thyroid, Graves' disease, Thermal ablation, Retrospective study, Efficacy assessment

## Abstract

**Background:**

Ultrasound-guided thermal ablation (TA) has emerged as a robust therapeutic approach for treating solid tumors in multiple organs, including the thyroid. Yet, its efficacy and safety profile in the management of Graves’ Disease (GD) remains to be definitively established.

**Methods:**

A retrospective study was conducted on 50 GD patients treated with TA between October 2017 and December 2021. Key metrics like thyroid volume, volume reduction rate (VRR), thyroid hormones, and basal metabolic rate (BMR) were evaluated using paired Wilcoxon tests.

**Results:**

The intervention of ultrasound-guided TA yielded a statistically significant diminution in total thyroid volume across all postoperative follow-up intervals—1, 3, 6, and 12 months—relative to pre-intervention baselines (*p* < 0.001). The median VRR observed at these time points were 17.5%, 26.5%, 34.4%, and 39.8%, respectively. Euthyroid status was corroborated in 96% of patients at the one-year follow-up milestone. Transient tachycardia and dysphonia were observed in three patients, while a solitary case of skin numbness was noted. Crucially, no instances of enduring injury to the recurrent laryngeal nerve (RLN) were documented.

**Conclusions:**

Our investigation substantiates ultrasound-guided TA as a pragmatic, well-tolerated, and safe therapeutic modality for GD. It effectively improves symptoms of hyperthyroidism, engenders a substantial reduction in thyroid volume, and restores thyroid hormone and BMR to physiological levels. Given its favorable safety profile, enhanced cosmetic outcomes, and minimally invasive nature, ultrasound-guided TA is a compelling alternative to thyroidectomy for GD patients.

**Supplementary Information:**

The online version contains supplementary material available at 10.1186/s13044-024-00198-4.

## Introduction

Hyperthyroidism, characterized by the thyroid gland’s excessive secretion and synthesis of thyroid hormones, exerts detrimental effects on various physiological systems, including the nervous, circulatory, and digestive systems [1]. Such symptoms significantly impair patients’ quality of life. GD is the predominant cause of hyperthyroidism, accounting for up to 80% of cases in iodine-sufficient regions [2]. The estimated annual incidence of GD ranges from 20 to 50 per 100,000 individuals and continues to rise [3, 4].

Currently, the primary therapeutic options for GD include antithyroid drugs (ATDs), radioactive iodine (RAI) therapy, and thyroidectomy [5]. While ATDs can alleviate symptoms and enhance life quality, they are associated with a high recurrence rate of 50–60% and potentially severe complications such as agranulocytosis, drug-induced liver injury, and even liver failure [6]. RAI therapy offers a lower recurrence rate but may exacerbate Graves’ ophthalmopathy (GO), induce acute thyroiditis, and increase cancer risk [7]. Thyroidectomy provides a high cure rate but carries the risk of long-term adverse effects like hypothyroidism, RLN paralysis, and scarring [8, 9].

Ultrasound-guided TA has emerged as a promising alternative to traditional treatments. This technique employs an internally cooled radiofrequency catheter inserted into the target nodule under ultrasound guidance to generate heat through alternating current. This heat induces irreversible tissue damage, resulting in coagulation necrosis [10, 11]. TA offers several advantages over traditional treatments, including higher precision, minimal invasiveness, quicker recovery, and repeatability [12]. Compared to thyroidectomy, TA is less traumatic, more cosmetically appealing, and more cost-effective [13]. Additionally, TA avoids the complications associated with RAI therapy, such as worsening of GO and acute thyroiditis [14].

Despite these advantages, there is a paucity of research evaluating the efficacy and safety of ultrasound-guided TA specifically for GD. This study aims to fill this gap by conducting a retrospective cohort study of GD patients treated with TA at our center between October 2017 and December 2021. Our objective is to establish TA as a viable, safe, and effective treatment modality for GD, thereby providing a reference for personalized treatment plans.

## Methods

### Study design

This study was a single-center, retrospective investigation conducted in accordance with the Declaration of Helsinki and approved by the Institutional Review Board of Xiamen Medical University (Ethical Approval No. 2,023,052). All participants or their legal guardians provided written informed consent. Our study protocol entailed treating patients with GD using ultrasound-guided TA, with subsequent follow-up visits scheduled at 1, 3, 6, and 12 months after the procedure. The procedure was performed by Dr. Lixin Zhou, a thyroid surgeon with extensive experience in managing thyroid diseases.

### Recruited cohort

GD diagnosis was based on clinical manifestations of hyperthyroidism, diffuse thyroid enlargement, suppressed serum thyroid Stimulating Hormone (TSH) levels, and elevated serum thyroid hormone concentrations [15–18]. Additional diagnostic criteria included exophthalmos, pretibial myxedema, and elevated thyroid-stimulating hormone receptor antibodies (TRAb) levels. In this study, the measurement of TRAb levels served as a substitute for thyroid stimulating antibodies (TSAb) levels, both for the diagnosis and for the prognostic evaluation of GD.

Patients who met these diagnostic criteria were included in the study. Those who declined ATDs, RIT, or surgical intervention for various reasons—such as ATD treatment failure, reluctance for long-term medication, concerns about radioactivity, cosmetic considerations, or risk of postoperative hypothyroidism—were also eligible. In total, 50 out of 57 patients who met these criteria between October 2017 and December 2021 were included in the study, with seven lost to follow-up.

### Monitoring and follow-up

Clinical characteristics, preoperative symptoms, and postoperative complications were meticulously recorded. Thyroid volume was assessed post-procedure using ultrasound imaging, employing the ellipsoid volume formula [19]. The VRR was calculated using the formula [(V0-V1)/V0] x 100, where V0 and V1 represent baseline and post-ablation thyroid volumes, respectively. Descriptive statistics were used to summarize changes in various clinical parameters, including thyroid hormone levels and BMR, calculated using the Gale method [20].

### Equipment

Ultrasound-guided thermal ablation (TA) can be executed utilizing various modalities, including microwave ablation (MWA), radiofrequency ablation (RFA), or laser techniques. Microwave ablation (MWA) therapy is characterized by rapid inactivation of the target tissue with a larger scope, shorter ablation time, less operator fatigue, and a more manageable goal of near-total thyroid ablation. Radiofrequency ablation (RFA) is also used, but the time required to ablate the same volume of target tissue is longer than that of microwave, and laser ablation requires even more time. At our center, microwave and radiofrequency techniques are primarily used for TA. The portable color ultrasound M-Turb (Sono Sound) is employed for ultrasonic image acquisition and TA guidance. The probe for detecting external organs is a 4–9 MHz linear array probe. RFA uses an S-1500 radiofrequency therapy system (Shanghai Meide Medical Co., Ltd.); a radiofrequency catheter model 10-131181 is used, with an active tip of 5, 7, 10, or 15 mm according to the baseline nodule volume. MWA is performed using an ECO-100A1 microwave treatment instrument (YIGAO Microwave System Engineering Co., Ltd., Nanjing, Jiangsu Province, China) and an ECO-100AI3 superficial organ ablation needle (16 G, total length: 10 cm, microwave transmitter away from the shaft tip: 3 mm). The power is set at 35 W, and the adjustment range is 30–40 W. In this study, the selection between MWA and RFA was determined by the technological advancements and equipment availability at our facility during different time periods, rather than by patient-specific factors. This approach was integral to our methodology and is critical for understanding the context of our results. Specifically, from October 2017 to December 2019, our facility was equipped with and hence utilized MWA for treating patients. As our technological capabilities evolved, we transitioned to using RFA from January 2020 to December 2021.

### Procedural details

Before undergoing ultrasound-guided TA for hyperthyroidism, patients were treated with ATD to control symptoms of thyrotoxicosis. For patients with a heart rate above 90 beats/min, add β-adrenergic blockers. For patients with difficulty controlling thyrotoxicosis and taking ATD treatment and patients with second-degree thyroid enlargement and above, potassium iodide oral solution is added (Start taking Luger’s solution 14 days before the procedure: 3 drops/time, three times/day, add one drop daily; continue to take it for seven days after the procedure, reduce one drop daily to 9 drops/time and stop the drug) [16, 17, 21].

To ensure that patients are fully informed and able to choose the treatment plan independently, the procedure consent form must be signed by the patient prior to the procedure. Patients were advised to abstain from food and drink for at least six hours prior to the procedure. Prior to the procedure, a routine ultrasound measurement of thyroid size and Color Doppler Flow Imaging (CDFI) to show thyroid blood perfusion is performed, and the puncture route for TA is planned.

The patient is positioned supine with hyperextended neck. The surgical area is routinely disinfected and draped. Conventional ultrasound is used to determine the location, size, and vascular distribution of the thyroid. Layered infiltration anesthesia under ultrasound guidance using 0.5% lidocaine is performed to separate the thyroid from the pre-thyroid muscles, forming an isolation zone to block heat conduction and prevent damage to surrounding structures. The space behind the thyroid capsule is usually not isolated to avoid tearing and bleeding caused by forced separation due to adhesion of the thyroid to the surrounding large blood vessels. A surgical incision is made in the midline of the neck at the projection of the thyroid isthmus. Under ultrasound guidance, the tip of the puncture needle is inserted into the thyroid, penetrating one side of the gland through the isthmus. The path is from the anterior and inner side next to the trachea, about 0.3 to 0.5 cm, puncturing to the deep part of the thyroid. The needle tip approaches the dorsal capsule of the gland but does not penetrate the thyroid capsule. The gland is ablated from inside to outside in a stepwise manner. During puncture, the needle tip is always kept within the ultrasound-visible range. The preset power range is 30 to 40 W. The thyroid is considered as multiple sections, starting from the middle part of the thyroid and ablating layer by layer upwards and downwards. Each layer uses an ablating sequence from inside to outside, deep to shallow. During ablation, a normal gland thickness of about 0.3 to 0.5 cm near the trachea should be preserved to avoid ablating the danger triangle area and protect the recurrent laryngeal nerve. The Moving-shot ablation technique is used, which can achieve near-total ablation of the hyperthyroid gland. The Moving-shot technique is used to ablate the glands sequentially from the inside to the outside until a gas response with hyperechoic gas is observed [22, 23]. After satisfactory ablation on one side, communication with the patient is made to confirm that there is no change in voice, then the same anesthesia and ablation treatment is applied to the other side of the thyroid. For patients with thick isthmus tissue, ablation of the thyroid isthmus is performed to complete near-total ablation treatment of both lobes and the isthmus of the thyroid in one go.

The ablation range is equivalent to the range of near-total thyroidectomy, only preserving a thin layer of glandular tissue on the inner posterior sides of the trachea, namely the danger triangle area [24], with a thickness of about 0.3 cm, measuring approximately 1.0 cm × 1.5 cm, about 3 g of thyroid tissue. This ensures the safety of important organs such as neck nerves, blood vessels, trachea, and parathyroid glands while achieving near-total thyroid ablation.

Following the TA procedure, the patient’s neck is protected from burns by applying compression ice while pressure is applied to stop bleeding and alleviate discomfort. On the first day after the procedure, the patient is closely monitored for complications such as fever, bleeding, dyspnea, cough, hoarseness, and thyroid storm. Additionally, thyroid function is tested during this time. After successful observation, the patient is discharged on the second day after the procedure.

To maintain thyroid function and manage any residual thyrotoxicosis, patients are treated with ATD, β-adrenergic blockers, and potassium iodide oral solution after the procedure [25]. It is important to note that the potassium iodide solution should be discontinued within ten days after the procedure [26]. Treatment with these drugs is typically maintained for approximately three months, after which they are gradually discontinued once symptoms of thyrotoxicosis have resolved and thyroid function has returned to normal levels. Three months after the TA, patients continued exhibiting elevated thyroid hormone levels, paired with suppressed thyroid-stimulating hormone levels. Even upon cessation of ATDs, these individuals maintained high levels of thyroid hormones and diminished TSH levels. The tapering of ATD dosage presented a challenge in preserving normal thyroid hormone levels. The above patients necessitate a secondary TA of the residual thyroid tissue.

In patients co-diagnosed with GD and active GO, prophylactic glucocorticoids are prescribed to curtail the advancement of GO post-TA and to address any ophthalmic complications that may arise postoperatively.

### Statistical analysis

Enumeration data were presented as frequencies and rates, while measurement data were expressed as medians ± one quartile. The Fisher exact test was employed to evaluate dichotomous variables between populations. The significance of the difference in the means of continuous variables between patients who underwent RFA and MWA was assessed via a two-tailed unpaired Wilcoxon test. A two-tailed paired Wilcoxon test was performed to compare the measurements before and after the procedure. The ggplot package was utilized to generate visualizations of thyroid volume changes. A p-value less than 0.05 was considered statistically significant. Statistical analysis was conducted using R statistics software (version 4.2.1).

## Results

### Clinical information

In our retrospective cohort study, we assessed a total of 50 patients diagnosed with GD, all of whom underwent ultrasound-guided TA. The patients were divided into two treatment groups: 29 received RFA, accounting for 58% of the cohort, and 21 underwent MWA, making up the remaining 42%. Notably, the majority of the study population was female, with 41 out of 50 patients (82%), aligning with the gender distribution reported in recent literature on GD [27]. Prior to the TA procedure, various measures were taken to stabilize the patient’s cardiac rhythm. Specifically, 12 patients (24%) were prescribed propranolol. Additionally, the ATD methimazole (MMI) was administered to 34 patients (68%) as a preoperative measure (Table 1).

**Table 1:** The clinical features of patients. Differences between the RFA and MWA groups are tested using the Wilcoxon and Fisher tests. TA, thermal ablation; RFA, radiofrequency ablation; MWA, microwave ablation; ATD, antithyroid drugs

	TA (*n* = 50)	RFA (*n* = 29)	MWA (*n* = 21)	P-value
Age (year)	41 (36-49.25)	41 (34.5–46.5)	43 (37.5–50.5)	0.34
Gender				
Male (%)	9 (18.0)	5 (17.2)	4 (19.0)	
Female (%)	41 (82.0)	24 (82.8)	17 (81.0)	1.00
Propranolol (%)	12 (24.0)	6 (20.7)	6 (28.6)	0.74
Pre-procedure ATD				
Methimazole (%)	34 (68.0)	18 (62.1)	16 (76.2)	0.37
Duration of pre-procedure ATD (month)	21.1 (9.85-35)	15.6 (3.6–35)	23.8 (13.1-36.325)	0.20
Duration of application (minute)	47 (40-53.25)	51 (40.5–56.5)	43 (39.5–48)	0.02
Length of stay (day)	2 (2–3)	2 (2-2.5)	2 (2–3)	0.31
Multiple TA (%)	30 (60.0)	19 (65.5)	11 (52.4)	0.39
Multiple TA intervals (month)	8.2 (4.8-13.05)	8.4 (4.8–14.1)	7.2 (4-12.3)	0.21
Thyroid volume (cm^3^)	23.7 (14.5–36.7)	24.1 (16.9–41.2)	20.2 (12.4–33.7)	0.57

In terms of procedure duration, the median time for the RFA group was 47 min (IQR: 40-53.25), whereas for the MWA group, it was 43 min (IQR: 39.5–48). Upon statistical analysis, we identified a significant variance between the RFA and MWA groups concerning procedure duration (*p* = 0.02). However, the length of hospital stay did not significantly differ between the two groups, with a median of 2 days (IQR: 2–3; *p* = 0.31). Multiple procedures were performed on 19 patients in the RFA group (65.5%) and 11 in the MWA group (52.4%). Fisher’s exact test showed no significant difference between the two groups in the incidence of multiple procedures (*p* = 0.39). Similarly, the time interval between the first and last TA procedures did not significantly differ between the two groups, averaging 8.4 months (IQR: 4.8–14.1) for RFA and 7.2 months (IQR: 4-12.3) for MWA (*p* = 0.21) (Table 1).

Within the complete cohort, a subset of three patients underwent three TA sessions, whereas a larger group of 30 patients participated in two sessions. The most frequently observed symptoms following the procedure included palpitations in 8 patients (16%), weight loss in 5 patients (10%), and fatigue in 3 patients (6%). During the study period, the overall incidence of post-procedure complications was 20% (*n* = 10). Among the subgroups, the RFA group had a lower incidence of complications at 13.8% (*n* = 4), compared to 28.6% (*n* = 6) in the MWA group. The primary complications manifested as tachycardia in five patients (10%), transient dysphonia in another five (10%), and skin numbness around the oral and limb areas in two patients (4%). Post-procedure tachycardia was successfully managed with propranolol and generally resolved within a week. By the time of the one-year follow-up, 96% of participants had achieved a euthyroid state, substantiating the efficacy of the TA procedure. Among the five patients presenting with GO, only two exhibited active GO and were pre-emptively administered prophylactic glucocorticoids prior to undergoing TA. Subsequent to the TA procedure, no further progression of GO was observed in these patients (Tables 2 and 3).

**Table 2:** Pre-procedure symptoms. Differences between the RFA and the MWA groups are tested using the Fisher test. TA, thermal ablation; RFA, radiofrequency ablation; MWA, microwave ablation

	TA (%) (*n* = 50)	RFA (%) (*n* = 29)	MWA (%) (*n* = 21)	P-value
Total	12 (24.0)	5 (17.2)	7 (33.3)	0.31
Palpitations	8 (16.0)	3 (10.3)	5 (23.8)	0.26
Ophthalmopathy	5 (10.0)	2 (6.9)	3 (14.3)	0.64
Heat intolerance	2 (4.0)	2 (6.9)	0 (0.0)	0.50
Anxiety	2 (4.0)	2 (6.9)	0 (0.0)	0.50
Weight loss	5 (10.0)	2 (6.9)	3 (14.3)	0.64
Fatigue	3 (6.0)	1 (3.4)	2 (9.5)	0.57
Dysphagia	2 (4.0)	2 (6.9)	0 (0.0)	0.50
Globus	1 (2.0)	1 (3.4)	0 (0.0)	1.00
Voice issues	0 (0.0)	0 (0.0)	0 (0.0)	1.00
Pressure	2 (4.0)	2 (6.9)	0 (0.0)	0.50

**Table 3:** Post-procedure complications. Differences between the RFA and the MWA groups are tested using the Fisher test. TA, thermal ablation; RFA, radiofrequency ablation; MWA, microwave ablation; RLN, recurrent laryngeal nerve

	TA (%) (*n* = 50)	RFA (%) (*n* = 29)	MWA (%) (*n* = 21)	P-value
Total	10 (20.0)	4 (13.8)	6 (28.6)	0.29
Tachycardia	5 (10.0)	4 (13.8)	1 (4.8)	0.38
Hematoma	0 (0.0)	0 (0.0)	0 (0.0)	1.00
Skin Numbness	2 (4.0)	0 (0.0)	2 (9.5)	0.17
Hypocalcemia (Transient/Permanent)	0 (0.0)	0 (0.0)	0 (0.0)	1.00
Hypoparathyroidism (Transient/Permanent)	0 (0.0)	0 (0.0)	0 (0.0)	1.00
Transient Dysphonia	5 (10.0)	1 (3.4)	4 (19.0)	0.30
Permanent RLN injury	0 (0.0)	0 (0.0)	0 (0.0)	1.00

### Thyroid volume

**Fig. 1:**
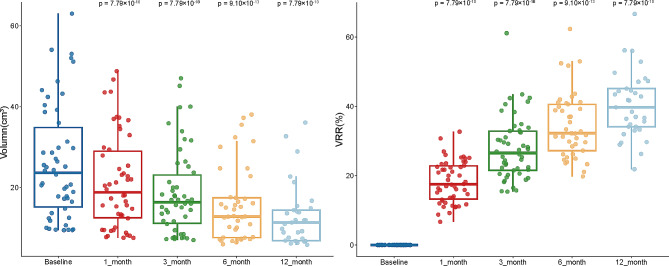
Volumetric reduction after thyroid thermal ablation. Box plots showing the variation of thyroid volume (**A**) and VRR (**B**) over pre-procedure and follow-up as medians ± one quartile, with the whiskers extending from the hinge to the smallest or largest value within 1.5 IQR from the box boundaries. The sample sizes of TA are 50, 50, 41, and 33, respectively. Differences between the two groups are tested using paired Wilcoxon test

The study monitored 50 patients over a 12-month period, carefully tracking the changes in thyroid gland volume. At baseline, the median thyroid volume stood at 23.7 cm³ (IQR: 14.5–36.7). Following the ultrasound-guided TA, we observed a substantial decrease in thyroid volume at multiple time points. Specifically, the median volume reduced to 18.8 cm³ (IQR: 11.8–29.5), 16.3 cm³ (IQR: 10.6–24.1), 14.8 cm³ (IQR: 9.1–22.9), and 13.3 cm³ (IQR: 8.6–18.9) at 1-, 3-, 6-, and 12-months post-procedure, respectively. Furthermore, we calculated the post-procedure thyroid VRR to quantify the effectiveness of the treatment. The VRR was 17.5% (IQR: 13.0–23.0), 26.5% (IQR: 21.3–32.9), 34.4% (IQR: 28.1–41.2), and 39.8% (IQR: 33.2–47.1) at 1-, 3-, 6-, and 12-months post-procedure, respectively (Fig. 1 and Tables S1 and S2). The data clearly illustrate a consistent and statistically significant reduction in thyroid volume over successive time points, substantiating the efficacy of TA in treating GD. Importantly, all patients reported relief from neck compression symptoms during the follow-up period, further validating the therapeutic benefits of the procedure. Additional radial data relating to thyroid volume can be found in Supplementary Tables S3-S5.

### Thyroid hormones

Pre-procedure assessments revealed elevated levels of T3, T4, and TRAb in all patients, with median values of free triiodothyronine (FT3): 13.500 pmol/L (IQR: 11.688–16.248), free triiodothyronine (FT4): 31.380 pmol/L (IQR: 27.118–35.637), and TRAb: 30.855 IU/L (IQR: 20.970-36.573). Concurrently, the TSH levels were markedly lower than those in healthy individuals, with a median value of TSH: 0.003 mIU/L (IQR: 0.003–0.003). Likewise, there were considerable reductions in thyroid hormone levels, supporting that TA effectively modulates thyroid function. One-month post-procedure, we observed a significant increase in TSH levels to a median of 2.439 mIU/L (IQR: 0.531–3.805; *p* = 7.75 × 10^− 10^). By the one-year mark, TSH levels had normalized, with a median value of 3.011 mIU/L (IQR: 2.130–4.291; *p* = 7.77 × 10^− 10^). Nevertheless, we observed a marked elevation in TRAb levels one month following the surgical intervention (*p* = 3.80 × 10^− 10^). Subsequently, both TRAb and thyroid hormone levels declined, reaching the normal physiological level (*p* = 9.93 × 10^− 10^). This confirms that ultrasound-guided TA has a long-term positive impact on thyroid function (Table 4, S6, and S7).

**Table 4:** The change of the thyroid hormones after TA (*n* = 50). Differences between the two groups are tested using the paired Wilcoxon tests. TA, thermal ablation; TSH, thyroid stimulating hormone; FT3, free triiodothyronine; FT4, free triiodothyronine; TRAb, Thyroid-stimulating hormone receptor antibodies

	TSH (mIU/L)	P_value	FT3 (pmol /L)	P_value	FT4 (pmol /L)	P_value	TRAb (IU/L)	P_value
Pre-procedure	0.003 (0.003–0.003)		13.500 (11.688–16.248)		31.380 (27.118–35.637)		30.855 (20.970-36.573)	
Post-procedure 1 month	2.439 (0.531–3.805)	7.75 × 10^− 10^	3.790 (2.069–4.675)	7.79 × 10^− 10^	15.147 (10.313–20.605)	7.79 × 10^− 10^	35.345 (25.515–45.829)	3.80 × 10^− 12^
Post-procedure 3 months	2.325 (1.101–3.940)	1.14 × 10^− 9^	3.815 (1.902–5.490)	7.79 × 10^− 10^	13.903 (10.095–18.863)	7.79 × 10^− 10^	22.622 (13.200-32.571)	3.32 × 10^− 5^
Post-procedure 6 months	2.795 (1.922–4.477)	1.10 × 10^− 9^	4.833 (3.216–6.340)	1.15 × 10^− 9^	17.260 (9.520-19.813)	7.79 × 10^− 10^	3.504 (1.320–8.460)	7.78 × 10^− 10^
Post-procedure 12 months	3.011 (2.130–4.291)	7.77 × 10^− 10^	3.543 (2.177–5.148)	7.79 × 10^− 10^	14.898 (8.375–20.228)	7.79 × 10^− 10^	1.896 (0.882–8.828)	9.93 × 10^− 10^

### Basal metabolic rate

To ensure patient safety during the procedure, medications such as ATDs, propranolol, and potassium iodide were administered to regulate heart rate and cardiac pressure. The Basal Metabolic Rate (BMR) was deliberately lowered to below 20% prior to the procedure as a precautionary measure against peri-procedural risks. Our data showed a significant drop in BMR levels at 1, 3, 6, and, 12 months post-procedure, aligning with changes in thyroid hormones and corroborating the therapeutic efficacy of TA for managing GD. In summary, our study provides compelling evidence for the efficacy and safety of ultrasound-guided TA in the treatment of GD, offering a promising alternative to traditional surgical interventions (Table 5 and S8).

**Table 5:** The change of BMR after TA (*n* = 50). Differences between the two groups are tested using the paired Wilcoxon test. TA, thermal ablation; RFA, radiofrequency ablation; MWA, microwave ablation

	TA (*n* = 50)	P_value	RFA (*n* = 29)	P_value	MWA (*n* = 21)	P_value
Pre-procedure	16.5 (13-18)		17 (13.5–18)		16 (11-19)	
Post-procedure 1 month	6 ((-0.25)-10)	8.06 × 10^− 9^	7 (1-10.5)	1.51 × 10^− 5^	4 ((-3)-10)	1.41 × 10^− 4^
Post-procedure 3 months	7 (3-10.25)	1.39 × 10^− 8^	8 (4-10)	7.52 × 10^− 6^	7 ((-1)-11)	5.49 × 10^− 4^
Post-procedure 6 months	6.5 (3.75-10)	8.74 × 10^− 9^	8 (4.5–11.5)	6.73 × 10^− 6^	4 ((-1)-8.5)	2.60 × 10^− 4^
Post-procedure 12 months	7.5 (3-11.25)	1.11 × 10^− 8^	8 (4-11.5)	7.25 × 10^− 6^	5 ((-1.5)-11.5)	3.86 × 10^− 4^

## Discussion

Hyperthyroidism is a complex medical condition characterized by excessive secretion and synthesis of thyroid hormones that manifests through the thyroid gland’s excessive secretion and synthesis of thyroid hormones. This hormonal imbalance has far-reaching implications, affecting a multitude of physiological systems, such as the nervous, circulatory, and digestive systems. A wide array of symptoms manifest, including but not limited to tachycardia, tremors in the extremities, significant weight loss, and specialized conditions like GO and thyroid dermopathy. These symptoms present not just medical challenges but also severely hamper the patient’s overall quality of life [27]. Within this complex landscape, GD stands out as a particularly challenging autoimmune form of hyperthyroidism. In GD, TSAb bind to the TSH receptor, setting off a cascade of events that result in uncontrolled synthesis and secretion of thyroid hormones [17].

The traditional therapeutic landscape for managing hyperthyroidism has largely been dominated by long-term treatments involving ATDs. Another longstanding approach has been RAI therapy, which uses radiation to selectively damage thyroid follicular cells, thereby inhibiting excessive hormone synthesis. Our research posits ultrasound-guided TA as a groundbreaking, minimally invasive alternative to conventional surgical thyroidectomy. One of the most striking findings was the significantly shorter duration of the TA procedure, with a median time of 47 min (IQR: 40-53.25). This stands in stark contrast to the traditional thyroidectomy, which requires a considerably longer average duration of around 123.2 ± 43.0 min [21]. It is worth mentioning that by the end of the one-year follow-up period, 96% of the participants had achieved a euthyroid state, with no patients exhibiting hypothyroidism or requiring levothyroxine replacement, further substantiating the long-term efficacy of the TA procedure. This demonstrates its potential as an effective and enduring alternative treatment for hyperthyroidism.

Our findings indicate that TA procedures are correlated with a reduced incidence of postoperative complications when contrasted with traditional surgical techniques [21]. This is likely due to the procedure being performed within the confines of the thyroid capsule, thus limiting the risk of injury to the parathyroid glands and other adjacent tissues. Although patients reported transient soft tissue edema, likely due to the use of 1% lidocaine, these symptoms generally resolved within 48 h, making them a manageable side effect.

Although the TA procedure successfully reduced thyroid gland volume and increased the VRR, certain patients expressed lingering aesthetic concerns, as outlined in Fig. 1 and Tables S1 and S2. Unlike surgical interventions, which remove the enlarged thyroid gland entirely, TA only reduces the gland’s size, leaving some patients dissatisfied with the immediate aesthetic outcome. Nevertheless, TA treatment significantly reduced the levels of thyroid hormones in patients, bringing them to normal levels. For instance, our data revealed an initial surge in TRAb levels following the surgical procedure, which was subsequently followed by a statistically significant decline. This suggests that the necrotic tissue resulting from TA may have a lower immunogenicity, potentially reducing the chances of exacerbated autoimmune responses (Table 4, S6 and S7). Furthermore, TA stabilized the patients’ BMR, which was consistent with the changes observed in thyroid hormones (Tables 4 and 5).

Ultrasound-guided TA offers several advantages in terms of safety, particularly in minimizing the risk of injury to surrounding tissues. One advantage of ultrasound guidance is that it effectively prevents direct damage to the surrounding skin, trachea, blood vessels, and nerves by the active tip. Another benefit is that the isolation zone established by 0.5% lidocaine in the interstitial space can avoid thermal damage caused by heat conduction to adjacent tissues [28]. However, it is crucial to acknowledge that TA, like any medical procedure, comes with its own set of risks, including potential post-procedure complications such as bleeding and voice changes due to RLN injury.

Compared with ATD and RAI, TA has numerous advantages, including no need for long-term medication, low recurrence rate, no blood cell deficiency, and no drug-induced liver injury. In addition, pregnant or breastfeeding patients can also use TA without the risk of exacerbating GO. However, there are still some disadvantages of TA, such as a broader range of injuries and the risk of post-procedure complications, including bleeding, voice changes caused by recurrent laryngeal nerve injury, parathyroid injury, and thyroid storm.

Compared with surgery, TA has several advantages, including cosmetic benefits, minor trauma, quick post-procedure recovery, low cost, fewer post-procedure complications, and a low incidence of hypothyroidism [29]. However, there may be a higher recurrence rate of hyperthyroidism. Additionally, TA still has some limitations and issues, such as the lack of medium and long-term follow-up data from large cohorts, the inability to address the cosmetic issue of an enlarged thyroid gland immediately, and the specific risk of adjacent tissue damage during the procedure. The amalgamation of imaging data from diverse sources like CT scans, MRI, contrast-enhanced ultrasonography, and (CDFI) could provide a more comprehensive evaluation of TA’s effectiveness and scope [30], thus reducing the incidence of multiple ablations. MRI assessment of thyroid volume changes before and after TA helps evaluate thyroid volume changes. Contrast-enhanced ultrasonography can better assess the perfusion of thyroid tissue before and after the procedure to evaluate the efficacy of TA. However, our department has yet to carry out a contrast-enhanced ultrasound project, an area for future research.

Despite the excellent short-term efficacy of TA, the long-term efficacy remains unknown. There is still a lack of large-sample, multi-center, and prospective clinical studies on TA treatment. Furthermore, the inclusion and diagnosis criteria and treatment standards need to be standardized to improve the accuracy and reliability of the research.

## Conclusions

Our study accentuates the high efficacy, safety, and tolerability of ultrasound-guided TA as a therapeutic approach for managing GD-induced hyperthyroidism. Notably, TA demonstrates marked efficacy in symptom alleviation, a significant reduction in thyroid gland volume, and the successful normalization of thyroid hormone levels and BMR. When juxtaposed against conventional surgical thyroidectomy, TA appears to offer several advantages, including reduced physical trauma, fewer post-procedure complications, and enhanced aesthetic outcomes. As such, ultrasound-guided TA emerges as a compelling alternative to surgical interventions for hyperthyroidism patients. Nevertheless, it is crucial to recognize that a knowledge gap still exists concerning the long-term efficacy of TA treatment. Therefore, future research should prioritize large-scale, multi-center, prospective clinical trials aimed at standardizing diagnostic criteria and treatment protocols for TA in hyperthyroidism management.

### Electronic supplementary material

Below is the link to the electronic supplementary material.

Supplementary Material 1

## Data Availability

The data used in this study will be made available upon request to the corresponding author. Access to the data will be subject to signing a data use agreement and approval from the institutional review board. Requests for data should be directed to zhoulix318@163.com.

## References

[1] Milo T, Korem Kohanim Y, Toledano Y, Alon U (2023). Autoimmune thyroid diseases as a cost of physiological autoimmune surveillance. Trends Immunol.

[2] Song Y, Wang X, Ma W, Yang Y, Yan S, Sun J (2023). Graves’ disease as a driver of depression: a mechanistic insight. Front Endocrinol (Lausanne).

[3] Smith TJ, Hegedüs L (2016). Graves’ Disease. N Engl J Med.

[4] McLeod DS, Cooper DS (2012). The incidence and prevalence of thyroid autoimmunity. Endocrine.

[5] Sjölin G, Holmberg M, Törring O, Byström K, Khamisi S, de Laval D (2019). The long-term outcome of treatment for Graves’ hyperthyroidism. Thyroid.

[6] El Kawkgi OM, Ross DS, Stan MN (2021). Comparison of long-term antithyroid drugs versus radioactive iodine or surgery for Graves’ disease: a review of the literature. Clin Endocrinol (Oxf).

[7] Ciarallo A, Rivera J (2020). Radioactive Iodine Therapy in differentiated thyroid Cancer: 2020 update. AJR Am J Roentgenol.

[8] Zhang D, Park D, Sun H, Anuwong A, Tufano R, Kim HY (2019). Indications, benefits and risks of transoral thyroidectomy. Best Pract Res Clin Endocrinol Metab.

[9] Guo Z, Yu P, Liu Z, Si Y, Jin M (2013). Total thyroidectomy vs bilateral subtotal thyroidectomy in patients with Graves’ diseases: a meta-analysis of randomized clinical trials. Clin Endocrinol (Oxf).

[10] Chu KF, Dupuy DE (2014). Thermal ablation of tumours: biological mechanisms and advances in therapy. Nat Rev Cancer.

[11] Han W, Liu F, Liu G, Li H, Xu Y, Sun S (2023). Research progress of physical transdermal enhancement techniques in tumor therapy. Chem Commun (Camb).

[12] Kim JH, Baek JH, Lim HK, Ahn HS, Baek SM, Choi YJ (2018). 2017 thyroid radiofrequency ablation Guideline: Korean society of thyroid Radiology. Korean J Radiol.

[13] Chen Z, Zhang W, He W (2023). Ultrasound-guided thermal ablation for papillary thyroid microcarcinoma: a systematic review. Clin Endocrinol (Oxf).

[14] Li HX, Xiang N, Hu WK, Jiao XL (2016). Relation between therapy options for Graves’ disease and the course of Graves’ ophthalmopathy: a systematic review and meta-analysis. J Endocrinol Invest.

[15] Bautista-Orduno KG, Dorsey-Trevino EG, Gonzalez-Gonzalez JG, Castillo-Gonzalez DA, Garcia-Leal M, Raygoza-Cortez K et al. American thyroid association guidelines are inconsistent with Grading of Recommendations Assessment, Development, and Evaluations-A meta-epidemiologic study. J Clin Epidemiol. 2020;123:180-8.e2. 10.1016/j.jclinepi.2020.02.010 32145366

[16] Ross DS, Burch HB, Cooper DS, Greenlee MC, Laurberg P, Maia AL (2016). 2016 American Thyroid Association Guidelines for Diagnosis and management of hyperthyroidism and other causes of thyrotoxicosis. Thyroid.

[17] Kahaly GJ, Bartalena L, Hegedüs L, Leenhardt L, Poppe K, Pearce SH (2018). 2018 European thyroid Association Guideline for the management of Graves’ hyperthyroidism. Eur Thyroid J.

[18] National Institute for Health and Care Excellence. Guidelines. Thyroid disease: assessment and management. London: National Institute for Health and Care Excellence (NICE) Copyright © NICE 2019.; 2019.

[19] Brunn J, Block U, Ruf G, Bos I, Kunze WP, Scriba PC. [Volumetric analysis of thyroid lobes by real-time ultrasound (author’s transl)]. Deutsche medizinische Wochenschrift (1946). 1981;106(41):1338-40. 10.1055/s-2008-1070506 7274082

[20] Henry CJ (2005). Basal metabolic rate studies in humans: measurement and development of new equations. Public Health Nutr.

[21] Frank ED, Park JS, Watson W, Chong E, Yang S, Simental AA (2020). Total thyroidectomy: safe and curative treatment option for hyperthyroidism. Head Neck.

[22] Ha EJ, Baek JH, Lee JH (2014). Moving-shot versus fixed electrode techniques for radiofrequency ablation: comparison in an ex-vivo bovine liver tissue model. Korean J Radiol.

[23] Lin WC, Tai YF, Chen MH, Luo SD, Huang F, Chen WC et al. Ultrasound-guided moving Shot Radiofrequency ablation of Benign Soft tissue neoplasm. Med (Kaunas). 2021;57(8). 10.3390/medicina57080830 PMC8402204 34441036

[24] Park HS, Baek JH, Park AW, Chung SR, Choi YJ, Lee JH (2017). Thyroid radiofrequency ablation: updates on innovative devices and techniques. Korean J Radiol.

[25] Piantanida E (2017). Preoperative management in patients with Graves’ disease. Gland Surg.

[26] Calissendorff J, Falhammar H (2017). Lugol’s solution and other iodide preparations: perspectives and research directions in Graves’ disease. Endocrine.

[27] Wiersinga WM, Poppe KG, Effraimidis G (2023). Hyperthyroidism: aetiology, pathogenesis, diagnosis, management, complications, and prognosis. Lancet Diabetes Endocrinol.

[28] Chung SR, Baek JH, Choi YJ, Lee JH (2019). Management strategy for nerve damage during radiofrequency ablation of thyroid nodules. Int J Hyperthermia: Official J Eur Soc Hyperthermic Oncol North Am Hyperth Group.

[29] Jin H, Lin W, Lu L, Cui M (2021). Conventional thyroidectomy vs thyroid thermal ablation on postoperative quality of life and satisfaction for patients with benign thyroid nodules. Eur J Endocrinol.

[30] Orloff LA, Noel JE, Stack BC, Russell MD, Angelos P, Baek JH (2022). Radiofrequency ablation and related ultrasound-guided ablation technologies for treatment of benign and malignant thyroid disease: an international multidisciplinary consensus statement of the American Head and Neck Society Endocrine Surgery Section with the Asia Pacific Society of thyroid surgery, Associazione Medici Endocrinologi, British Association of Endocrine and thyroid surgeons, European Thyroid Association, Italian Society of endocrine surgery units, Korean Society of Thyroid Radiology, latin American thyroid Society, and Thyroid Nodules Therapies Association. Head Neck.

