# Supplementary material for: Efficacy and safety of ultrasound-guided thermal ablation of graves’ disease: a retrospective cohort study
Source: Thyroid Res. 2024 Jun 3;17:10. doi: 10.1186/s13044-024-00198-4 (PMC11145836; doi:10.1186/s13044-024-00198-4)
Supplement: Supplementary file 1 — Supplementary Material 1 [file 13044_2024_198_MOESM1_ESM.docx]

| **Supplementary Table 1.** Differences in total volume (cm^3^) after TA. Differences between the two groups are tested using the paired Wilcoxon test. TA, RFA, and MWA sample sizes are 50/50/41/33, 29/29/23/18, and 21/21/18/15, respectively. TA, thermal ablation; RFA, radiofrequency ablation; MWA, microwave ablation. | | | | | | |
| --- | --- | --- | --- | --- | --- | --- |
|  | TA (n = 50) | P-value | RFA (n = 29) | P-value | MWA (n = 21) | P-value |
| Pre-procedure | 23.7 (14.5-36.7) |  | 24.1 (16.9-41.2) |  | 20.2 (12.4-33.7) |  |
| Post-procedure 1 month | 18.8 (11.8-29.5) | 7.79X10^-10^ | 19.5 (13.1-33.7) | 7.79X10^-10^ | 18.0 (9.5-28.7) | 7.79X10^-10^ |
| Post-procedure 3 months | 16.3 (10.6-24.1) | 7.79X10^-10^ | 16.0 (12.3-30.6) | 7.79X10^-10^ | 16.7 (8.4-21.9) | 7.79X10^-10^ |
| Post-procedure 6 months | 14.8 (9.1-22.9) | 9.10X10^-13^ | 15.0 (11.4-25.9) | 9.10X10^-13^ | 14.6 (7.5-20.1) | 9.10X10^-13^ |
| Post-procedure 12 months | 13.3 (8.6-18.9) | 7.79X10^-10^ | 13.6 (10.4-22.9) | 7.79X10^-10^ | 12.9 (6.8-17.3) | 7.79X10^-10^ |

| **Supplementary Table 2.** Total volume reduction rate after TA. Differences between the two groups are tested using the paired Wilcoxon test. TA, RFA, and MWA sample sizes are 50/50/41/33, 29/29/23/18, and 21/21/18/15, respectively. TA, thermal ablation; RFA, radiofrequency ablation; MWA, microwave ablation. | | | | | | | |
| --- | --- | --- | --- | --- | --- | --- | --- |
|  | TA (%) (n = 50) | P-value | RFA (%) (n = 29) | P-value | MWA (%) (n = 21) | | P-value |
| Pre-procedure | 0.0 |  | 0.0 |  | 0.0 (21) |  | |
| Post-procedure 1 month | 17.5 (13.0-23.0) | 7.79X10^-10^ | 17.4 (13.8-22.1) | 7.79X10^-10^ | 20.0 (11.5-24.0) | 7.79X10^-10^ | |
| Post-procedure 3 months | 26.5 (21.3-32.9) | 7.79X10^-10^ | 24.6 (21.6-31.0) | 7.79X10^-10^ | 30.2 (20.4-36.8) | 7.79X10^-10^ | |
| Post-procedure 6 months | 34.4 (28.1-41.2) | 9.10X10^-13^ | 31.7 (26.7-39.9) | 9.10X10^-13^ | 39.8 (31.3-43.4) | 9.10X10^-13^ | |
| Post-procedure 12 months | 39.8 (33.2-47.1) | 7.79X10^-10^ | 39.7 (32.4-44.5) | 7.79X10^-10^ | 44.3 (34.0-52.0) | 7.79X10^-10^ | |

| **Supplementary Table 3.** The change of the thyroid diameter after TA (n = 50). The diameter is measured in millimeters. Differences between the two groups are tested using the paired Wilcoxon test. TA, RFA, and MWA sample sizes are 50/50/41/33, 29/29/23/18, and 21/21/18/15, respectively. TA, thermal ablation; RFA, radiofrequency ablation; MWA, microwave ablation; VRR, volume reduction rate. | | | | | | | | | | |
| --- | --- | --- | --- | --- | --- | --- | --- | --- | --- | --- |
|  | Left_Length | Left_width | Left_thickness | Right_Length | Right_width | Right_thickness | Volume (ml) | P-value | VRR (%) | P-value |
| Pre-procedure | 63 (50-71) | 26 (20.75-31) | 20 (13.8-27) | 53.5 (46.5-61.3) | 23.5 (20-27.3) | 18 (12-23.3) | 23.7 (14.5-36.7) |  | 0 (0-0) |  |
| Post-procedure 1 month | 50 (43-56) | 21.5 (17-26.3) | 16.5 (11.8-20.3) | 59 (47-67.3) | 24 (18.8-28.3) | 18.5 (13-26) | 18.8 (11.8-29.5) | 7.79X10^-10^ | 17.5 (13.0-23.0) | 7.79X10^-10^ |
| Post-procedure 3 months | 56 (44.75-64.5) | 22.5 (18.5-27) | 17 (12-24.3) | 46.5 (41.8-52.8) | 20.5 (18-25) | 16 (10-20) | 16.3 (10.6-24.1) | 7.79X10^-10^ | 26.5 (21.3-32.9) | 7.79X10^-10^ |
| Post-procedure 6 months | 45 (40-51) | 19 (17-24) | 15 (9.5-18) | 55 (44.5-64) | 21 (15.5-25.5) | 15 (11-23) | 12.8 (7.6-17.5) | 9.10X10^-13^ | 32.2 (26.7-40.7) | 9.10X10^-13^ |
| Post-procedure 12 months | 54 (42-61.5) | 20 (15.5-24.5) | 14 (10-18) | 43 (38.5-50.5) | 19 (16-23.5) | 12 (9-16.5) | 11.3 (6.8-14.6) | 7.79X10^-10^ | 39.7 (33.7-46.1) | 7.79X10^-10^ |
| **Supplementary Table 4.** The change of the thyroid diameter after RFA (n = 29). The diameter is measured in millimeters. Differences between the two groups are tested using the paired Wilcoxon test. TA, RFA, and MWA sample sizes are 50/50/41/33, 29/29/23/18, and 21/21/18/15, respectively. TA, thermal ablation; RFA, radiofrequency ablation; MWA, microwave ablation; VRR, volume reduction rate. | | | | | | | | | | |
|  | Left_Length | Left_width | Left_thickness | Right_Length | Right_width | Right_thickness | Volume (ml) | P-value | VRR (%) | P-value |
| Pre-procedure | 49 (42-63.5) | 23 (20-27) | 20 (15.5-25.5) | 65 (52-72) | 24 (17.5-29) | 22 (17.5-28) | 24.1 (16.9-41.2) |  | 0 (0-0) |  |
| Post-procedure 1 month | 61 (48.5-68.5) | 22 (16.5-26.5) | 20 (16.5-26) | 46 (39.5-58.5) | 21 (17.5-24.5) | 19 (15.5-24) | 19.5 (13.1-33.7) | 3.73X10^-9^ | 17.4 (13.8-22.1) | 3.73X10^-9^ |
| Post-procedure 3 months | 60 (48-67) | 21 (15.5-26.5) | 20 (16-25.5) | 44 (37.5-55.5) | 20 (17.5-23.5) | 18 (13.5-23) | 16.0 (12.3-30.6) | 3.73X10^-9^ | 24.6 (21.6-31.0) | 3.73X10^-9^ |
| Post-procedure 6 months | 42 (36-54) | 19 (17-23) | 16 (12-21) | 60 (46-65) | 20 (13-24) | 19 (15-24) | 12.9 (10.2-17.6) | 2.38X10^-7^ | 30.8 (26.2-38.8) | 2.38X10^-7^ |
| Post-procedure 12 months | 57.5 (42-63.25) | 20 (13.75-24.25) | 17 (12.25-22) | 41 (36.25-52.5) | 19 (16.8-22.3) | 15 (8.8-17.5) | 11.7 (8.5-15.0) | 7.63X10^-6^ | 39.1 (33.2-43.9) | 7.63X10^-6^ |
| **Supplementary Table 5.** The change of the thyroid diameter after MWA (n = 21). The diameter is measured in millimeters. Differences between the two groups are tested using the paired Wilcoxon test. TA, RFA, and MWA sample sizes are 50/50/41/33, 29/29/23/18, and 21/21/18/15, respectively. TA, thermal ablation; RFA, radiofrequency ablation; MWA, microwave ablation; VRR, volume reduction rate. | | | | | | | | | | |
|  | Left_Length | Left_width | Left_thickness | Right_Length | Right_width | Right_thickness | Volume (ml) | P-value | VRR (%) | P-value |
| Pre-procedure | 54 (49-66.5) | 29 (21.5-32.5) | 16 (13-25.5) | 55 (50.5-58.5) | 25 (19-30) | 13 (11.5-19.5) | 20.2 (12.4-33.7) |  | 0 (0-0) |  |
| Post-procedure 1 month | 50 (46-62) | 26 (20-31) | 16 (12-22.5) | 52 (47-54.5) | 24 (16.5-28.5) | 13 (10-19.5) | 18.0 (9.5-28.7) | 9.54X10^-7^ | 20.0 (11.5-24.0) | 9.54X10^-7^ |
| Post-procedure 3 months | 48 (43-60) | 25 (20-28) | 15 (11.5-21.5) | 48 (45-51.5) | 23 (18-26.5) | 12 (10-18) | 16.7 (8.4-21.8) | 9.54X10^-7^ | 30.2 (20.4-36.8) | 9.54X10^-7^ |
| Post-procedure 6 months | 50.5 (41.8-60.5) | 24 (18-28) | 12.5 (11-15) | 47 (42.75-50) | 22 (16.5-26.3) | 10 (9-18) | 11.4 (7.3-18.8) | 7.63X10^-6^ | 34.1 (30.0-43.0) | 7.63X10^-6^ |
| Post-procedure 12 months | 51 (41-58) | 24 (18-25) | 12 (10-14) | 47 (41-49) | 21 (14-25) | 11 (9-14) | 8.9 (6.8-14.9) | 6.10X10^-5^ | 41.8 (34.1-48.0) | 6.10X10^-5^ |

| **Supplementary Table 6.** The change of the thyroid hormones after RFA (n = 29). Differences between the two groups are tested using the paired Wilcoxon test. RFA, radiofrequency ablation; TSH, thyroid stimulating hormone; FT3, free triiodothyronine; FT4, free triiodothyronine; TRAb, Thyroid-stimulating hormone receptor antibodies. | | | | | | | | |
| --- | --- | --- | --- | --- | --- | --- | --- | --- |
|  | TSH (mIU/L) | P-value | FT3 (pmol /L) | P-value | FT4 (pmol /L) | P-value | TRAb (IU/L) | P-value |
| Pre-procedure | 0.003 (0.003-0.003) |  | 13.610 (11.600-17.280) |  | 29.230 (26.160-33.470) |  | 29.490 (16.960-34.910) |  |
| Post-procedure 1 month | 1.908 (0.345-3.704) | 2.69X10^-6^ | 3.680 (1.650-4.569) | 3.73X10^-9^ | 17.840 (13.012-21.363) | 3.73X10^-9^ | 32.933 (16.973-47.780) | 5.10X10^-7^ |
| Post-procedure 3 months | 1.907 (1.118-2.905) | 4.00X10^-6^ | 2.640 (1.690-5.970) | 3.73X10^-9^ | 13.421 (10.000-15.780) | 2.70X10^-6^ | 26.894 (13.763-33.759) | 4.86X10^-2^ |
| Post-procedure 6 months | 3.301 (2.270-4.408) | 3.83X10^-6^ | 5.210 (3.412-6.450) | 4.00X10^-6^ | 17.230 (9.520-19.840) | 2.70X10^-6^ | 3.110 (1.320-7.980) | 3.73X10^-9^ |
| Post-procedure 12 months | 2.752 (0.523-4.400) | 2.69X10^-6^ | 4.356 (3.090-5.160) | 3.73X10^-9^ | 16.150 (9.880-21.665) | 3.73X10^-9^ | 1.430 (0.878-5.588) | 1.12X10^-8^ |
| **Supplementary Table 7.** The change of the thyroid hormones after MWA (n = 21). Differences between the two groups are tested using the paired Wilcoxon test. MWA, microwave ablation; TSH, thyroid stimulating hormone; FT3, free triiodothyronine; FT4, free triiodothyronine; TRAb, Thyroid-stimulating hormone receptor antibodies. | | | | | | | | |
|  | TSH (mIU/L) | P-value | FT3 (pmol /L) | P-value | FT4 (pmol /L) | P-value | TRAb (IU/L) | P-value |
| Pre-procedure | 0.003 (0.003-0.003) |  | 13.490 (12.030-15.240) |  | 32.180 (27.630-36.451) |  | 31.870 (29.454-38.454) |  |
| Post-procedure 1 month | 2.956 (1.989-3.923) | 6.40X10^-5^ | 4.180 (2.290-6.343) | 9.54X10^-7^ | 10.770 (7.290-16.240) | 9.54X10^-7^ | 37.570 (28.427-43.028) | 9.54X10^-6^ |
| Post-procedure 3 months | 3.121 (1.095-4.408) | 6.40X10^-5^ | 4.732 (3.800-5.460) | 9.54X10^-7^ | 17.780 (10.380-21.570) | 9.54X10^-7^ | 17.577 (12.332-28.766) | 6.68X10^-5^ |
| Post-procedure 6 months | 2.393 (1.663-4.500) | 6.35X10^-5^ | 4.690 (2.410-5.900) | 9.54X10^-7^ | 17.290 (11.330-19.730) | 9.54X10^-7^ | 5.800 (1.425-14.030) | 9.54X10^-7^ |
| Post-procedure 12 months | 3.586 (2.748-4.265) | 9.54X10^-7^ | 2.180 (1.562-5.110) | 9.54X10^-7^ | 13.460 (6.860-15.980) | 9.54X10^-7^ | 4.440 (1.085-12.160) | 9.54X10^-7^ |

| **Supplementary Table 8.** The change of vital signs after TA (n = 50). Blood pressure is measured in millimeters of mercury. Differences between the two groups are tested using the paired Wilcoxon test. TA, thermal ablation; BMR, basal metabolic rate. | | | | | |
| --- | --- | --- | --- | --- | --- |
| TA (n = 50) | | | | | |
|  | Pulse (bpm) | Systolic pressure | Diastolic pressure | BMR (%) | P-value |
| Pre-procedure | 82.5 (75.25-86.25) | 123 (113.75-132) | 74 (70.75-84.25) | 16.5 (13-18) |  |
| Post-procedure 1 month | 78 (75-81.25) | 117 (111.5-121) | 77.5 (71-83) | 6 ((-0.25)-10) | 8.061X10^-9^ |
| Post-procedure 3 months | 78.5 (72-83.25) | 112.5 (106-119) | 75 (68-80) | 7 (3-10.25) | 1.385X10^-8^ |
| Post-procedure 6 months | 79.5 (72-83) | 115 (109-120) | 75 (69.75-80) | 6.5 (3.75-10) | 8.735X10^-9^ |
| Post-procedure 12 months | 76 (72-83) | 114 (105.75-119.25) | 74 (67.25-77) | 7.5 (3-11.25) | 1.114X10^-8^ |
| RFA (n = 29) | | | | | |
|  | Pulse (bpm) | Systolic pressure | Diastolic pressure | BMR (%) | P-value |
| Pre-procedure | 82 (70-87) | 125 (113.5-132) | 73 (70-83) | 17 (13.5-18) |  |
| Post-procedure 1 month | 78 (76-81.5) | 117 (112-121) | 78 (71-83.5) | 7 (1-10.5) | 1.51X10^-5^ |
| Post-procedure 3 months | 81 (73.5-84) | 112 (106-119.5) | 75 (69-80) | 8 (4-10) | 7.52X10^-6^ |
| Post-procedure 6 months | 81 (74-84) | 115 (105.5-120) | 75 (68.5-80) | 8 (4.5-11.5) | 6.73X10^-6^ |
| Post-procedure 12 months | 79 (73-83.5) | 114 (105-119.5) | 73 (68-77) | 8 (4-11.5) | 7.25X10^-6^ |
| MWA (n = 21) | | | | | |
|  | Pulse (bpm) | Systolic pressure | Diastolic pressure | BMR (%) | P-value |
| Pre-procedure | 85 (77.5-86.5) | 122 (112-132.5) | 75 (71-87) | 16 (11-19) |  |
| Post-procedure 1 month | 78 (74-81.5) | 117 (106.5-121) | 77 (71.5-82.5) | 4 ((-3)-10) | 1.41X10^-4^ |
| Post-procedure 3 months | 76 (70.5-82.5) | 113 (107-119) | 75 (63.5-79.5) | 7 ((-1)-11) | 5.49X10^-4^ |
| Post-procedure 6 months | 73 (71.5-82) | 115 (110.5-120.5) | 76 (72-80.5) | 4 ((-1)-8.5) | 2.60X10^-4^ |
| Post-procedure 12 months | 72 (69.5-83) | 114 (107-120.5) | 75 (64.5-77.5) | 5 ((-1.5)-11.5) | 3.86X10^-4^ |
